# A tEMTing target? Clinical and experimental evidence for epithelial-mesenchymal transition in the progression of cutaneous squamous cell carcinoma (a scoping systematic review)

**DOI:** 10.1007/s12672-022-00510-4

**Published:** 2022-06-06

**Authors:** Benjamin Genenger, Jay R. Perry, Bruce Ashford, Marie Ranson

**Affiliations:** 1 School of Chemistry and Molecular Bioscience, University of Wollongong, Wollongong, NSW Australia; 2 Illawarra Health and Medical Research Institute, Wollongong, NSW Australia; 3 School of Medicine, University of Wollongong, Wollongong, NSW Australia

**Keywords:** Epithelial-mesenchymal transition, Cutaneous squamous cell carcinoma, Metastasis, Targeted therapy, Urokinase plasminogen activator system, Systematic review, UV-induced

## Abstract

Cutaneous squamous cell carcinoma (cSCC) is a disease with globally rising incidence and poor prognosis for patients with advanced or metastatic disease. Epithelial-mesenchymal transition (EMT) is a driver of metastasis in many carcinomas, and cSCC is no exception. We aimed to provide a systematic overview of the clinical and experimental evidence for EMT in cSCC, with critical appraisal of type and quality of the methodology used. We then used this information as rationale for potential drug targets against advanced and metastatic cSCC. All primary literature encompassing clinical and cell-based or xenograft experimental studies reporting on the role of EMT markers or related signalling pathways in the progression of cSCC were considered. A screen of 3443 search results yielded 86 eligible studies comprising 44 experimental studies, 22 clinical studies, and 20 studies integrating both. From the clinical studies a timeline illustrating the alteration of EMT markers and related signalling was evident based on clinical progression of the disease. The experimental studies reveal connections of EMT with a multitude of factors such as genetic disorders, cancer-associated fibroblasts, and matrix remodelling via matrix metalloproteinases and urokinase plasminogen activator. Additionally, EMT was found to be closely tied to environmental factors as well as to stemness in cSCC via NFκB and β-catenin. We conclude that the canonical EGFR, canonical TGF-βR, PI3K/AKT and NFκB signalling are the four signalling pillars that induce EMT in cSCC and could be valuable therapeutic targets. Despite the complexity, EMT markers and pathways are desirable biomarkers and drug targets for the treatment of advanced or metastatic cSCC.

**Graphical Abstract:**

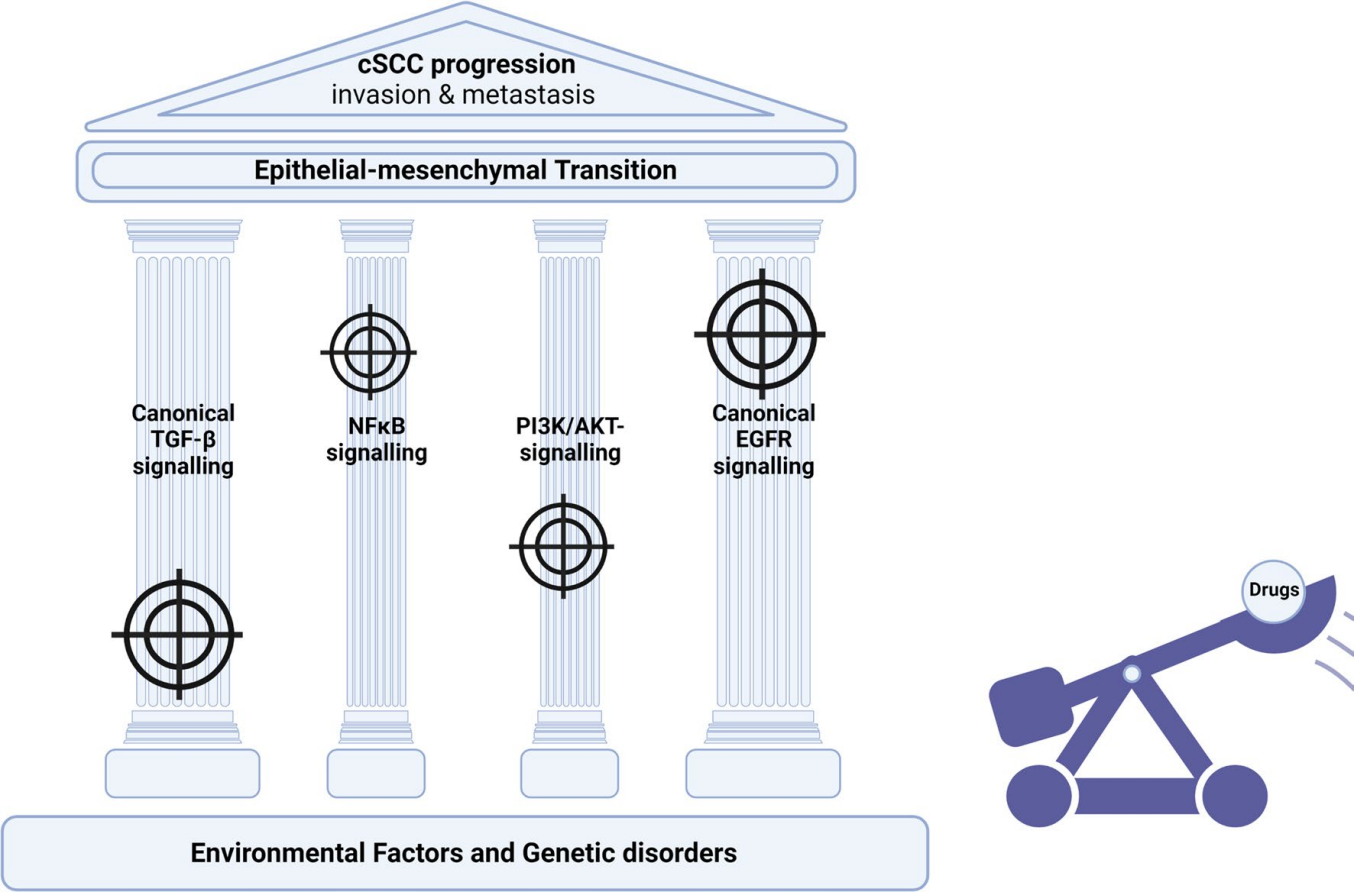

**Supplementary Information:**

The online version contains supplementary material available at 10.1007/s12672-022-00510-4.

## Introduction

### Rationale and background

Epithelial Mesenchymal Transition (EMT) is a process involved in tissue development, fibrosis, and cancer progression [1]. Evidence for the involvement of EMT during the invasive and metastatic process has been found in multiple carcinomas including head and neck squamous cell carcinoma (HNSCC) [2], pancreatic carcinoma [3], gastric cancer [4], and non-small cell lung cancer [5]. EMT has implications in therapy response and resistance to conventional chemotherapy and radiotherapy [6]. An increasing body of evidence also provides mechanistic links between EMT and immunosuppression in the tumor microenvironment (TME) [2, 7, 8]. Consequently, EMT markers were found to correlate with response to immunotherapy [9] and innate resistance to immunotherapy [10]. Additionally, the relevance of EMT markers as biomarkers for metastasis and for more accurate risk stratification of primary tumors is under investigation [11–14]. This notion is further supported by the fact that cells along the metastatic cascade such as at the invasive front [15], and circulating tumor cells present EMT markers [14].

The role of EMT in cutaneous squamous cell carcinoma (cSCC) pathogenesis is not well defined despite an increasing global incidence of cSCC. Since 1990, deaths attributed to non-melanoma skin cancer (NMSC) have more than doubled to reach 65,000 deaths world-wide in 2017 [16]. Despite constituting only 20 percent of NMSC cases, cSCC accounts for roughly 70 percent of all NMSC-associated deaths [17]. Risk factors, progression and therapeutic interventions for cSCC have been subject to multiple reviews [17–20]. Briefly, Patient-specific risk factors for cSCC include age, sex, skin type, and immunosuppression [19, 21]. Ultra violet (UV) radiation exposure, immunosuppression and chronic arsenite exposure are major environmental risk factors for cSCC [22, 23]. Additionally, inherited conditions such as xeroderma pigmentosum or epidermolysis bullosa (EB), specifically Kindler syndrome and recessive dystrophic EB (RDEB), favour the formation of cSCC [24, 25]. However, UV-induced cSCC is the most prevalent aetiology and is characterised by C to T (and CC > TT) transitions and a high tumor mutational burden [26].

Clinically, UV-induced precursor lesions, known as actinic keratosis (AK), progress to cSCC [17]. A model of progression via two different pathways, the classical or differentiated pathway has been pioneered by Fernandez-Figueras et al. [27] and was adapted by other reviewers [28]. The classical pathway involves full-thickness keratinocyte atypia (Bowen’s disease, cSCC in situ) prior to the acquisition of invasive properties (cSCC) and is in line with a more conservative progression model [29]. During the more aggressive differentiated pathway, atypical keratinocytes located only in the lower epidermal layers invade the underlying stroma. Locally advanced disease and metastatic disease are characterized by an increasing loss of differentiation and high mortality rates [18, 30]. Early-stage cSCC can be removed surgically with a curative rate greater than 90 percent [31]. However, recurrent cSCC, locally advanced (lacSCC), and metastatic disease (mcSCC) are often unresectable and associated with poor prognosis and significant morbidity [31–33]. Currently, there are no reliable biomarkers to predict metastasis of primary disease and the underlying mechanisms are understudied [23]. While immunotherapy is an emerging treatment for lacSCC and mcSCC with significant clinical benefits [34, 35], there are currently no reliable therapeutic biomarkers predicting response of cSCC to immunotherapy [35]. Due to its epithelial origin, the involvement of EMT in therapy resistance including resistance to immunotherapy, invasion, and metastasis of cSCC is plausible and the subject of ongoing research.

### Objectives

We aimed to provide a systematic review of the nature, extent, and quality of the evidence, from both experimental (in vitro and xenograft models) and clinical studies examining EMT in cSCC progression. This included a critical appraisal of the cell line-based models, markers and assays used to study EMT in cSCC based on the EMT International Association (TEMTIA) guidelines for experimental studies on EMT [36]. Their statement provides definitions to unify terminology as well as guidelines for cell line-based studies to adequately support claims of observed EMT. Researchers are urged to underpin findings of EMT in a combinatorial approach involving both EMT markers (e.g. E-cadherin, cytokeratins, integrins, vimentin), as well as changes in cellular properties (e.g. loss of cell–cell interactions, increased motility, decreased adhesion). The statement further highlights the complex non-linear nature of the process and delineates EMT from linked but distinct processes such as differentiation, stemness, survival, and metabolism. The statement concludes with the importance of EMT heterogeneity in tumor progression and the metastatic cascade and outlines the implications as a therapeutically targetable process. For clinical studies, all studies investigating at least one epithelial and mesenchymal marker or underlying signalling molecules with experimental validation were considered. In clinical research, the expression of EMT markers is sufficient as a proof for EMT due to the inherent link with the invasive biology of cancer. Based on our synthesis of information describing the basic signalling pathways driving EMT in cSCC progression and past efforts of modulating EMT in cSCC therapeutically, this review also evaluated the potential for targeting EMT as a valid therapeutic strategy for lacSCC and mcSCC.

## Methods

This review was planned and conducted in accordance with the Preferred Reporting Items for Systematic Reviews and Meta-analysis extension for Scoping Reviews (PRISMA-ScR) [37]. A complete checklist is attached as Supplementary File 1. This study has not been registered in databases for the registration of systematic literature reviews. However, to avoid duplicates, Open Science Framework, PROSPERO, and Cochrane databases were checked for similar protocols and studies prior to commencement and submission of the work.

All primary literature on studying the role of EMT in the progression of cSCC were considered regardless of aetiology. English and peer-reviewed studies published before 26/10/2021 were included if they provided clinical data or experimental data generated using human-derived models such as cell lines or xenograft models. Murine models were excluded as they represent either a different disease aetiology (i.e. chemically induced models), different species of origin, and an extensive body of literature beyond the scope of this review.

The search was conducted using PubMed and Medline (EBSCOhost) as subject specific databases as well as Scopus and Web of Science as general databases. Epithelial-Mesenchymal Transition, Cell plasticity, and Skin neoplasms were identified as relevant Medical Subject Headings (MeSH). The search string for each database can be accessed in Supplementary File 2. The search results were exported and uploaded to the platform Rayyan.ai [38] where the duplicates were removed and the record screening was conducted. To ensure maximum coverage, the bibliography of relevant reviews were scanned for eligible studies.

For clinical investigations, the stage during the clinical progression, the EMT markers investigated, and observed tendencies were extracted and presented as Fig. 2. For studies using cell lines, information about the model used, location/aetiology of the respective model, entity investigated, as well as interventions or alterations made were identified. Additionally, the EMT markers, EMT-associated properties, and EMT-transcription factors (EMT-TFs) involved were charted. The extracted methodology was assessed for compliance with the guidelines on EMT research proposed by TEMTIA (Supplementary table 2) [36]. A study was rated compliant to the TEMTIA guidelines if it incorporates at least one epithelial marker (e.g. E-cadherin) and one mesenchymal marker (e.g. Vimentin, Fibronectin), as well as investigation of at least one EMT-related property (e.g. migration, invasion). The integration of EMT-TFs was optional. Limitations of this study include incomplete or insufficient indexing of manuscripts that may have prevented the identification of all possible eligible studies. Additionally, this study is subject to publication bias as drug candidates eliciting the opposite effect or no effect are unlikely to be published [39].

## Results

### Included studies

**Fig. 1:**
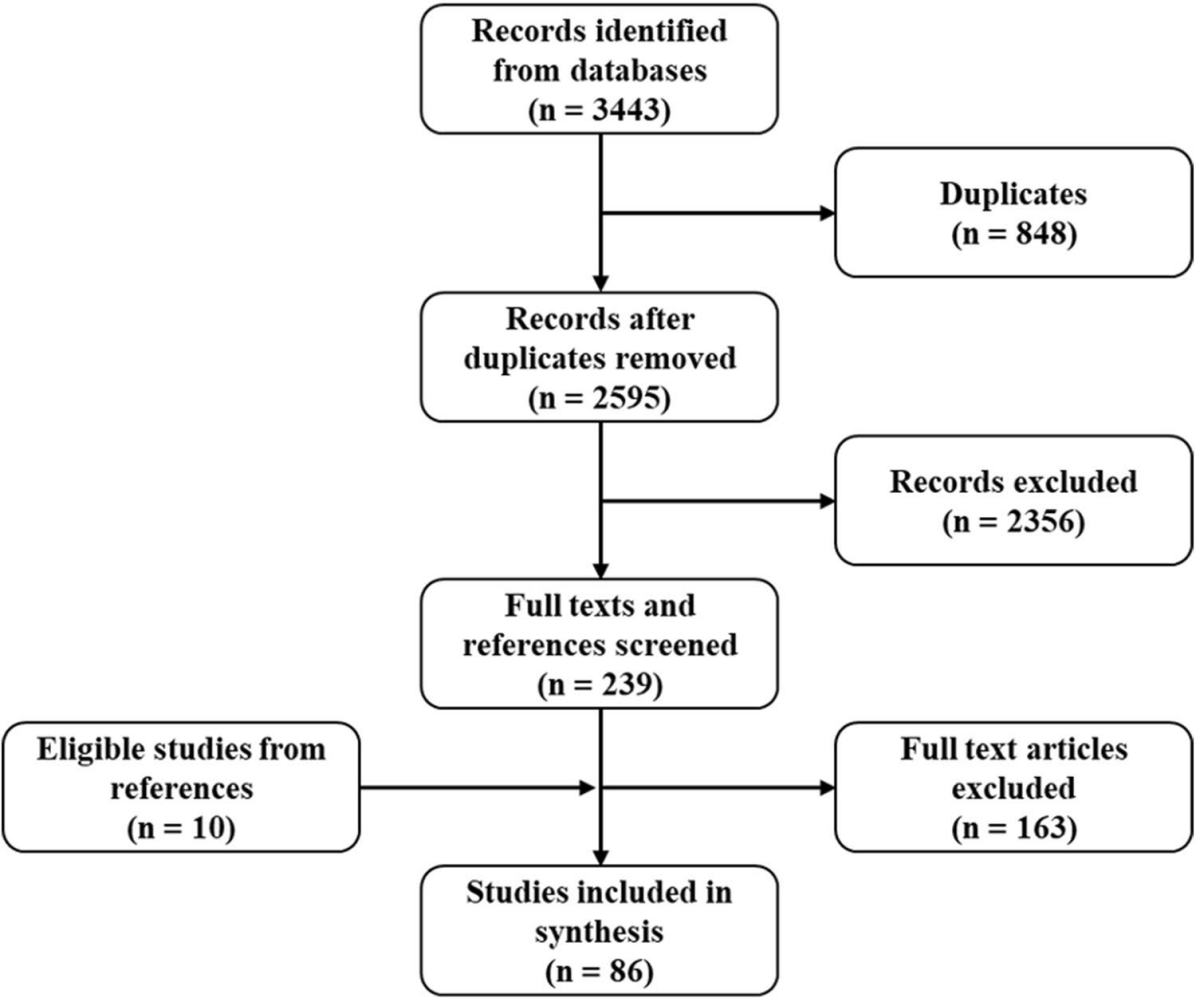
Selection of the sources of evidence

The screening of 3443 search results yielded 86 eligible studies (Fig. 1). Those excluded covered either a different disease (melanoma, BCC, cervical SCC, HNSCC), were secondary literature (review articles, book chapters), were unrelated to EMT, and/or used inducible murine models (DMBA, TPA, UV). Eligible studies encompassed 44 cell line-based (experimental) studies, 22 clinical studies, and 20 studies integrating both cell line work and clinical investigation (Supplementary Table 1 and 2).

### Characteristics of studies

Of the 64 studies encompassing cell-line models identified in the search (Supplementary Table 2), 79% were compliant with the guidelines suggested by TEMTIA and 13% provided insufficient evidence for their claims of EMT [33]. The remaining 8% were marked as not applicable due to study scope and design (e.g. differential gene expression analysis). However, investigation of spatial and/or transcriptional information beyond the pure protein levels of markers as obtained by Western Blotting was rare. In addition, integration of EMT-TFs and linking of specific transcription factors to their influences on EMT markers and properties in keratinocytes is infrequent.

While many of the experimental studies met TEMTIA guidelines**,** the cell line models used to investigate EMT in cSCC (Table 1) misrepresent the patient population and aetiology. The most frequently used model (40%) is the vulva-derived A431 cell line. The location of the primary tumor makes UV involvement (the most frequent cause of cSCC) in the aetiology unlikely, as has been noted by others [40]. Male sex is a risk factor for developing cSCC accounting for a 1.5- to twofold increase in incidence and increased mortality rates [41]. The two most frequently used models, A431 and SCC-13, are both derived from female donors and account for 57% of studies reversing the clinically observed male to female ratio. This may be of concern due to hormonal differences between the sexes and the potential role of hormonal regulation of EMT in cSCC [42–45].

**Table 1:** Cell line models used to study EMT in cSCC (used in > 5% of studies)

Model	Frequency of studies (%)	Location of tissue of origin/Patient characteristics
A431	40	Vulva (F, 85)
SCC-13	17	Face (F, 56)
HaCaT	15	Upper back/Transformed (As/ Ras) (M, 62)
MET1/2/4	14	hand/ hand/ left axillary lymph node (M, 45)
SCL1	9	Face, Caucasian (F, 74)
SCC12	9	Face (M, 60)
HSC-5	6	Location unknown/Japanese (M, 75)
NHEK/ PHK	6	Normal adult skin/Variable Location(HPV/ ectopic protein expression)
SCC-IC1	5	Right temple (M, 75)

M: male; F: female

There were no uniform set of markers used to assess EMT. A listing for each study specifying marker and methods is available in Supplementary Table 1 and 2. The most frequently employed epithelial and mesenchymal markers were E-cadherin, Cytokeratins, and EpCAM as well as Vimentin, N-Cadherin, Fibronectin and α-SMA actin, respectively. Sometimes other non-canonical EMT markers were employed to assess other closely linked properties or processes such as disassembly of cellular junctions (Girdin, α-/βcatenin, ZO1, Desmoglein 3), differentiation and stemness (KLF4, involucrin, CD133, CD44), and ECM remodelling (MMPs, uPAR, PAI-1). EMT marker analysis was primarily performed via Western blotting (84%), immunofluorescence (39%), and RT-qPCR (22%). Morphological changes towards a more spindle like phenotype are were used as starting point to prompt further investigation. Migration and invasion were frequently assessed using transwell migration assays, scratch wound assays, transwell migration assays into Matrigel coating, or organotypic assays. Cell adhesion was rarely evaluated but when done, used either a trypsinization assay or bead-based cell traction force assays. EMT property related assays were often paired with assays assessing stemness (colony/spheroid/tumor-forming assays) or proliferation (MTS/ MTT, CCK8, counting).

**Fig. 2:**
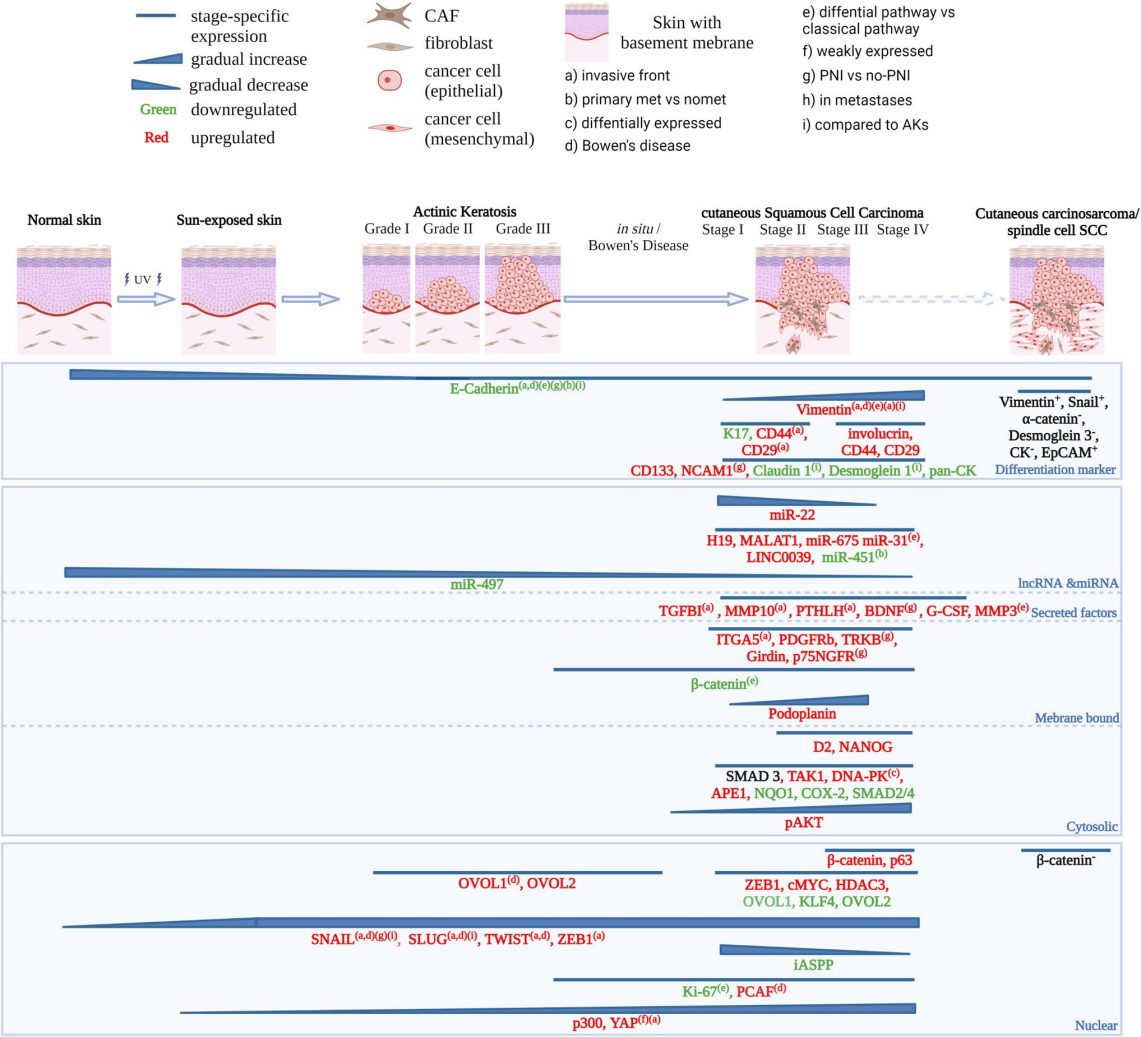
Summary of EMT markers and related signalling during the clinical progression of cSCC as determined through eligible studies included in this review (see supplementary table 1). In the progression of normal skin to cSCC the number of EMT markers steadily increases as the regulatory barriers fall and underlying pathways become activated. In cSCC the expression of EMT markers is by no means homogenous. Consequently, the contextual nature of the presented evidence needs to be considered (see footnotes). scSCC and CCS are rare extreme forms of cSCC often only reported on a case basis. Hence, + and − denote the quality of the immunohistochemical analysis for these disease states [76]

Of the many reports screened that included clinical samples, only 22 purely clinical investigational studies and 20 integrating both clinical investigation and cell line investigated EMT or related signalling in cSCC. A listing of each of these eligible studies specifying marker, methods and tissue comparisons is available in Supplementary 1. Again, as for experimental studies, the markers used to assess EMT were varied and included mRNA, proteins and miRNA/lncRNA expression analyses by comparison most often to normal or matched skin. Synthesis of this data in a timeline of cSCC progression is summarized in Fig. 2 and discussed below.

### Synthesis of results

#### Clinical progression of cSCC is paralleled by increased acquisition of a mesenchymal phenotype and EMT markers

Human epidermal keratinocytes display alterations of surface markers and transcription factors consistent with an EMT-phenotype early during the clinical progression. Bakshi et al. [46] reported reduced E-cadherin levels paralleled with increased Snail, Twist and Slug expression in sun-exposedskin (SES) vs non-sun-exposed skin (NSES). Others have found that miR-497 levels are significantly reduced in SES vs NSES. miR-497 is a negative modulator of the EMT markers Slug, N-cadherin and Vimentin, a direct repressor of *SERPINE1* and inhibitor of migratory properties in cSCC cell lines [47]. In cSCC, Snail and Slug levels are negatively correlated with E-cadherin levels [48–50].

While, an increasing body of evidence suggests that EMT is the determining factor for the progression of AK and Bowen’s disease to cSCC, differences between the classical and differentiated pathway have been reported. Saenz-Sardà et al. [51] found significantly lower expression of EMT markers Vimentin, E-cadherin, and membranous β-catenin in cSCC arising through the differentiated pathway when compared to the classical pathway. Additionally, the proliferation marker Ki67 was significantly increased in cSCC arising through the classical pathway [51]. Additional differences between the pathways include increased miR-31 and MMP levels in the differentiated pathway [52]. Nevertheless, the expression of markers at the invasive front of cSCC and Bowen’s disease acquiring de novo invasive ability suggest a pivotal role of the EMT process in facilitating invasion in cSCC [51, 53].

The importance of EMT in cSCC is further supported by increased expression of EMT-TFs and mesenchymal markers including Snail, Slug, ZEB1, Twist, Podoplanin, and Vimentin [46, 48, 49, 54–59] with increasing progression of the disease and increasing loss of differentiation. The gain of mesenchymal markers and properties is complemented by loss of epithelial markers (Involucrin, E-cadherin, KLF4, Cytokeratin, Claudin1) and apparent disassembly of cellular junctions [46, 53, 55–57, 60–68]. For example, Girdin, an adherence junction protein closely linked to E-Cadherin, has been associated with collective migration. Furthermore, Girdin expression is correlated to well-differentiated cSCC but is lost in poorly differentiated cSCC [69]. Toll et al. [58] linked cSCC expressing Vimentin, Twist, ZEB1, nuclear βcatenin and podoplanin to lymph node metastasis in a study cohort comprising tumors from 146 patients. Additionally, Vimentin levels correlated with recurrence, disease specific death, tumor stage, perineural invasion (PNI), desmoplasia, and differentiation. Podoplanin, despite not being a classical EMT-marker, was also identified by another independent study as predictor of regression-free survival [54]. Perineural invasion (PNI) is an established predictor of recurrence, metastasis, and poor prognosis in cSCC [70]. Brugière et al. [67] mapped cells with EMT-features to the site of PNI and increased neurotrophin signalling. This evidence links EMT to both lympho-vascular and PNI, two hallmarks of advanced disease and poor patient outcome.

The OVOL transcription factors 1 and 2 antagonize EMT-TFs and hence are important negative regulators of EMT [71]. OVOL1 and 2 protein levels are upregulated in benign precursor lesions of cSCC, such as AKs and Bowen’s disease (cSCC in situ) compared to cSCC [49, 50]. Compared to AKs, *OVOL1/2* expression is lost (or reduced) in cSCCs and this is associated with increased expression of EMT markers, Vimentin and Zeb1 [49]. Additionally, Murata [49] found a significant inverse association between OVOL2 and Zeb1 levels using 30 AK and 30 cSCC samples and increased *ZEB1* expression upon OVOL1 and OVOL2 knockdown in A431 cells. Furthermore, Ito et al. [50] report significantly reduced c-Myc levels and invasiveness in A431 cell upon OVOL1 knockdown. Interestingly. Ito et al. [50] could not confirm the loss OVOL2 with progression from Bowen’s disease to cSCC. Rather, they report OVOL2 translocation to the cytoplasm in cSCC, which hinders OVOL2 to act as a transcription factor. Altogether, these studies suggest a role of OVOL transcription factors as the last guardian against malignancy in pre-neoplastic lesions.

Ji et al. [15] resolved the spatial architecture of 10 cSCC with matched normal skin using a combination of single cell-RNA sequencing, spatial transcriptomics, and multiplexed ion beam imaging. They identified a subpopulation of keratinocytes exclusive to tumor tissue. These tumor-specific keratinocytes (TSKs) are located towards the invasive edge of the tumor and possess an EMT-like signature. TSKs express markers of EMT (Vimentin, ITGA5) despite lacking the expression of classical EMT-transcription factors (excluding *SLUG*). Single-cell regulatory network interference and clustering nominated AP1 and ETS transcription factors as regulators of EMT in TSKs. Additionally, their preferential localization at the invading edge infers invasive migratory properties when compared to their basal, differentiating and cycling counterparts. Furthermore, TSKs display a broad spectrum of EMT markers reflecting the high complexity and plasticity underlying the EMT continuum.

Even further along the epithelial-mesenchymal spectrum than cSCC are spindle cell cSCC (sc-cSCC), a poorly differentiated form of cSCC with a characteristic mesenchymal phenotype [72]. Nakamura et al. [62] reported a case of cSCC mimicking an atypical fibroxanthoma staining positive for Vimentin and Snail whilst staining negative for Cytokeratin. Iwata et al. [63] reported cases of sc-cSCC with complete loss of E-Cadherin, p120-catenin, and Desmoglein-3. More recently, Shimokawa et al. [61] showed increased nuclear staining of Snail, increased cytoplasmic Vimentin, and decreased levels of E-cadherin paralleled by significant reduction of COX2 levels in six sc-SCC compared to three non-sc-cSCC. Combined with the case reports, this warrants the consideration of sc-SCC as a tumor displaying advanced features of EMT and clinical progression of cSCC. Even more advanced, the position of cutaneous carcinosarcomas (CCS), biphasic tumors constituting of both a mesenchymal and epidermal component, in this clinical progression remains elusive [73]. The involvement of EMT in the formation of these tumors is up for debate [65, 74, 75]. Matching genotypes of both phases and EpCAM positive staining of mesenchymal and epidermal components both point towards singular epidermal origin of CCS and infer a quasi-full EMT as potential mechanism [65]. However fascinating, the rarity of sc-cSCC and CCS makes them less relevant in the overall picture [72, 73].

#### Genetic disorders associated with increased frequency of aggressive cSCC facilitate EMT via altered cell–matrix interactions

Epidermolysis bullosa (EB) is a group of conditions that are associated with early onset and rapid progression of cSCC [24, 77]. Kindler syndrome is caused by mutations in the *FERMT1* gene [78]. *FERMT1* codes for Kindlin-1, a protein co-localizing at focal adhesions and involved in the activation of their receptor functions. Lack of Kindlin-1 is associated with dysregulated integrin signalling, cell adhesion and migration [79]. Immortalized patient-derived kindlin-deficient keratinocytes display reduced cell–cell adhesion, cell–matrix adhesion, and epithelial markers. Conversely, induction of mesenchymal markers, ECM components and proteases point towards a more mesenchymal phenotype [80]. Conflicting data by Ji et al. [15] identifies *FERMT1* as an essential transducer of integrin signalling in TSKs and amplification across many cancers. Additional studies suggest a role for *FERMT1* in regulating EMT across multiple cancers including [81–83]. In cSCC, evidence points towards a contextual involvement of Kindlin-1 in EMT regulation and warrants further investigation [15, 80].

RDEB patients bear loss-of-function mutations in the *COL7A1* gene and produce highly aggressive and metastatic cSCC [77, 84]. Knockdown of ColVII in the non-RDEB cSCC cell lines, Met1 and SCC-IC1, increased migration and invasion by enhancing EMT and preventing differentiation [85]. In xenografts derived from ColVII knockdown SCC-IC1 cells, recombinant human ColVII reduced the effects of the ColVII deficiency. In addition to increased angiogenesis, ColVII knockdown resulted in amplified TGF-β1 signalling and upregulation of urokinase plasminogen activator (uPA), SERPINE1 and VEGFA [86]. Clinically, RDEB tumors displayed increased EMT markers and increased levels of TGFβR1. Furthermore, loss of ColVII directly correlated with decreased levels of involucrin, an epithelial differentiation marker in vivo [86]. Twaroski et al. [87] confirmed findings of increased TGF-β1 signalling and identified MEK/ERK, p38 and SMAD3 as downstream effectors and mediators of an EMT phenotype. Together these studies provide an attractive model for the high aggressiveness and high metastasis rates in RDEB cSCC.

#### Cancer-associated fibroblasts induce EMT in cSCC via paracrine growth factor, cytokine, integrin and MMP signalling 

Cancer-associated fibroblasts (CAFs) and other cancer-associated cell types in the tumor microenvironment are implicated in several cancers to induce EMT via paracrine signalling (refer to Fig. 2) [1, 15, 88]. Co-culture of fibroblast and cSCC cancer cells has been shown to be paralleled by increased CAF and EMT marker expression [89]. In cSCC, fibroblast subtype can also play a significant role in cancer progression. For example, reticular fibroblasts favour EMT and invasion compared to papillary fibroblasts [89]. On the same note, Bordignon et al. [90] reported different CAF subtypes with diametric influences on EMT markers and tumor invasiveness in cSCC. A TGF-β induced CAF population significantly increased invasiveness and tumorigenic expansion in vivo but not FGF2-induced CAFs. The increased aggressiveness of TGF-β induced CAFs was associated with increased EMT markers (Vimentin, Snail, Slug and Twist) and altered TME (e.g. COL1A1 secretion) in the tumor cells. Additional evidence, confirms that patient-derived fibroblasts from RDEB patients secrete TGF-β to facilitate EMT in RDEB-cSCC cell lines [87]. However, fibroblasts contribute to EMT not only via TGF-β secretion. For example, conditioned media derived from senescent fibroblasts induced EMT in post-senescent keratinocytes via MMP-PAR-signalling. Matching clinical evidence suggest that EMT-associated traits and markers such as gelatinolytic activity, PAR-1 expression, andTWIST expression were elevated in aged human skin samples [91]. This might provide another mechanistic link between the risk factor age and cSCC progression aside from the continuous accumulation of UV-induced mutations.

Clinically, Sasaki et al. [92] used independent clustering based on CAF and EMT-related markers to establish a significant correlation between clinic pathological subgroup and malignancy. Interestingly, the subgroups correlated with clinical parameters such as lymph node metastasis, tumor thickness and tumor size. Ji et al. [15] showed that TSKs engage in reciprocal signalling with CAFs, endothelial cells, macrophages, and myeloid-derived suppressor cells (MDSC). These interactions form a complex network of signalling molecules, including ECM components (*FN1*, *COL1A1*), cytokines (*TGFB1*, *TGFB3*, and *CXCL*s), growth factors (*PGF*, *VEGFA*), proteases (*MMP9*), and integrins (*ITGA3*, *ITGB1*). However, the influence of the individual factors on the EMT status remains subject to further investigation. For example, some evidence suggests, that stromal macrophages do not play a major role in the induction of EMT in cSCC [59].The tumor microenvironment alters the EMT status of tumor cells not only through paracrine signalling but also through the properties and composition of the ECM itself [93]. For example, HPV transformed-keratinocytes (N/TERT keratinocytes) elicit an EMT response to fibronectin via α3β1-integrins [94]. The influence of matrix stiffness and mechanotransduction pathways on EMT has been investigated in other cancers but remains underexplored in cSCC [95, 96].

#### β-catenin provides a mechanistic explanation for the close link between stem-like properties and EMT in cSCC

Cancer stem cells have gained traction in recent years as they link major challenges modern cancer therapy faces including recurrence, therapy resistance and metastasis [6, 97]. Even though stemness and EMT are separate phenomena, they are closely linked [6]. Cells, staining positive for the stem cell markers CD44 and CD29, at the invasive front of tumors also displayed characteristics consistent with EMT [98]. In three kidney organ transplant recipients, EMT markers (Vimentin, Slug, and Snail) were co-expressed in CD133 expressing (stem) cells in invasive areas of skin SCCs but not concomitant AKs or normal skin [60]. A mechanistic link is provided by the downregulation of E-cadherin, which releases sequestered β-catenin from the cell membrane to the nucleus [69, 99]. Furthermore, this might work synergistically with dysregulated Wnt/β-catenin signalling, an inducer of cancer stem cell properties [100–102]. Clinical evidence shows the co-localization of βcatenin and E-cadherin at cellular junctions [51, 69]. Loss of E-cadherin is associated with the disassembly of those junctions, decreased adherence and increased nuclear β-catenin (Fig. 2 and 4) [51, 69]. The transcriptional repression of E-cadherin is mediated by canonical EMT-TFs (e.g. Snail) as well as other transcription factors such as Grhl3 (Fig. 4) [61, 103]. Increased nuclear β-catenin has been observed in tumors with poor differentiation and correlated to lymph node metastasis [55, 58, 69].

The close link between stemness and EMT is supported by an increasing body of in vitro evidence. Biddle et al. [104] performed CD44/ EpCAM based sorting of the cell lines PM1, MET1 and MET2, derived from dysplastic skin, the primary lesion, and a recurrence at the same anatomical site, respectively [105]. They observed an increase of in the Epcam low/ CD44 high population with increasing malignancy. Further, a more prominent EMT phenotype, sphere forming ability but reduced proliferative ability distinguished the EpCAM low/CD44 high population from the EpCAM high/ CD44 high population. CD44/ITGB1 based sorting of the A431 cell line also identified a subset within the cancer stem cells (CSC) that display EMT characteristics. In murine xenograft models of A431 cells, the CD44 high/ ITGB1 high stem cells gave rise to significantly bigger and more aggressive tumors [98]. Additionally, the simultaneous regulation by common upstream regulators including ARMC8, ΔNp63α, p38/NFκB, transglutaminase II (TGA2) and Axl [106–111], as well as pharmaceutically active substances further tightens the link between of EMT and CSC properties [108, 112].

#### Proteases including the urokinase plasminogen activator system are underexplored markers and EMT effectors

Proteases such as matrix metalloproteases contribute to tumor cell invasion and EMT, via multiple mechanisms [113, 114]. Proteolytic cleavage of the ECM allows for increased migration and liberates latent signalling molecules such as EGF, HGF, and TGF-β [115, 116]. In two-dimensional A431 cell culture, broad inhibition of MMPs and MMP-9 knockdown reduced- EMT marker expression and motility. Additionally, the EMT-TF Snail induced expression of MMP-9 [117]. MMP-2 contributes to the invasiveness and migratory abilities in TGF-β-induced EMT of RDEB cells [87]. TGF-β1 and EGF treatment in Ras-transformed HaCaT-cells induced an EMT phenotype and increased collagen remodelling. The broad-spectrum inhibition of MMPs using GM6001 increased cell attachment and abrogated collagen degradation [118]. An often-overlooked contribution of MMPs to EMT signalling is mediated by G-protein-coupled cell surface receptors, transducing a signal upon MMP cleavage [119]. MMPs, MMP-1, MMP-2, and MMP-3, secreted by senescent fibroblasts induced EMT markers and migration via a PAR1-mediated mechanism [91].

Wilkins-Port et al. [118] identified the involvement of another protease system in TGF-β1 and EGF induced EMT of ras-transformed HaCaT- cells, the urokinase plasminogen activator system (uPAS). Inhibition of the uPAS using amiloride (uPA inhibitor) or plasminogen activator inhibitor 1 (PAI-1, encoded by SERPINE1), an endogenous uPA inhibitor, reduced MMP-1 and MMP-10 levels as well as attenuated collagen. Additionally, *PLAU* (uPA) is part of the TSK-gene signature identified by Ji et al. [15]. TGF-β1 and EGF induce the expression of PAI-1 and multiple studies report the transcriptional regulation of *SERPINE1* parallels the induction of EMT [47, 120]. Mizrahi et al. [47] found progressive down-regulation of miR-497 through promotor methylation during the progression of SES to cSCC. Expression of miR-497 reduced SERPINE1 levels as well as levels of other EMT related genes.

#### miRNA and lncRNA expression profile changes during EMT play a central role in its regulation

During the progression from normal skin to cSCC, the expression of many non-coding RNAs such as miRNAs and lncRNA are altered. miRNAs regulate the transcription of proteins by regulating the stability of their respective target mRNAs [121]. Mizrahi et al. [47] investigated the differential expression of miRNAs in a clinical progression of NSES to cSCC. The changes in the miRNAs expression ranged from gradual increase/decrease to stepwise acquisition/loss creating a progression specific profile. For example, the loss of the tumor suppressor miR-497 increased SMAD signalling, EMT as well as *SERPINE1* expression. Other studies also found miRNAs dysregulating some of the signalling pathways central to EMT including the PI3K/Akt pathway and the Wnt-pathway. The tumor suppressive miR-451a, a suppressor of PDK1, is downregulated in metastatic vs non-metastatic tumors [122]. The suppression of PTEN by miR-21 leads to increased pathway activity and Akt activation [123, 124]. The oncogenic miR-22 is gradually upregulated with increasing grade of cSCC and promotes stemness via Wnt- signalling [100]. A mechanistic study by Robinson et al. [125] identified miR-211 and miR-205 as part of an iASPP/ p63 epigenetic feedback loop regulating EMT, with the latter directly targeting Zeb1 and p63.

LncRNA can act as molecular sponges for miRNAs by competing for binding with their cognate mRNAs [126]. However, other mechanism of action such as chromatin remodelling, transcriptional regulation or mRNA post-transcriptional regulation are possible [121]. For example, the lncRNA HOTAIR sponges miR-326 leading to an increase of PRAF2 and a more prominent EMT phenotype [126]. MALAT1 is upregulated in cSCC compared to normal skin and promotes EMT via modulation of Wnt-signalling [127]. Clinically, Li et al. [128] found a correlation between the upregulation of LINC00319 and tumor size, lymphovascular invasion, and TNM stage. In cSCC cell lines, LINC00319 favoured migration, invasion, and EMT marker expression [128]. LncRNA, H19, and miR-675 are upregulated in cSCC. Upregulation of H19 increased miR-675 levels as well as the expression of EMT markers [129].

#### Environmental factors such as arsenite induce EMT and transformation by widespread alteration of miRNA expression, mRNA expression and induction of IL-6 signalling

**Fig. 3:**
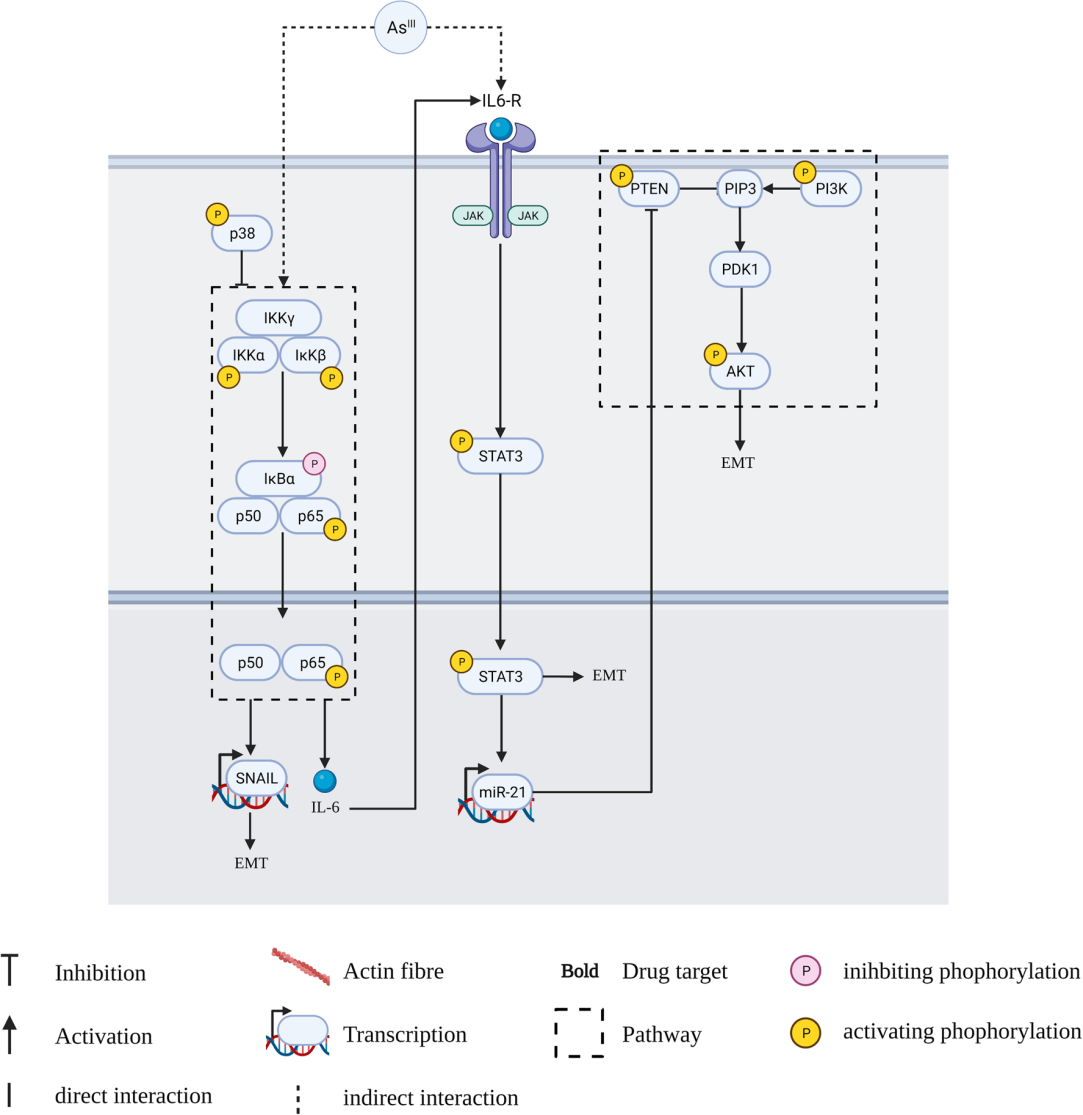
Signalling for arsenite-induced EMT. Arsenite activates NFκB signalling pathways, which induce EMT via Snail as well as lead to the secretion of IL-6. Autocrine IL-6 signalling induces EMT and activates PI3K signalling via miR-21 mediated repression of PTEN [76]

After UV exposure, arsenic exposure is one of the biggest occupational hazards for developing NMSC [130]. The acute and chronic toxicity of arsenite can be replicated in vitro by exposing HaCaT cells to arsenite (0.1–1 µM) for up to 28 weeks [124, 131]. Al-Eryani et al. [132] found a significant dysregulation of EMT and cell cycle genes as early as 7 weeks after exposure to arsenite. Banerjee et al. [131] reported decreases in ZO-1, a tight junction protein, and increased Slug after 19 weeks. After 28 weeks, the arsenic-exposed cells display an advanced EMT phenotype. Additionally, pathway analysis of the differentially expressed miRNAs and mRNAs showed inhibition of the ER stress pathway. Investigations into the molecular mechanism implicate NFκB, PI3K and IL-6 signalling as the major contributors to the EMT phenotype (Fig. 3). IL-6 induces miR-21 via STAT3 cytokine signalling [123]. In return, miR-21 activates Akt via the inhibition of PTEN [124]. Furthermore, the increased CSC-like properties are ascribed to increase p38/ NFκB signalling as well as the induction of IL-6 [107, 108]. Upregulation of the EMT-TF Snail provides a direct link to canonical EMT signalling [107, 108].

Other external factors such as UV and ROS also promote EMT in malignant keratinocytes. UV radiation induces the EMT transcription factor Snail via an AP1-dependent mechanism and UV exposure is associated with decreased E-cadherin levels clinically [46, 133]. Additionally, UV irradiation promotes cSCC via increased production of reactive oxygen species (ROS) [134]. Conversely, the tumor suppressor and negative modulator of oxidative stress, NAD(P)H dehydrogenase (NQO1), is lost in cSCC. Adenoviral expression of NQO1 reduced ROS levels and attenuated EMT [135]. On the other hand, cellular stress can induce autophagy by reducing the levels of the autophagy marker, p62 [136]. p62 directly binds and stabilizes the EMT-TF Twist1 [137]. Additionally, p62 can induce NFκB signalling further linking inhibition of autophagy to EMT [136].

#### Thyroid hormone and Estrogen signalling modulates EMT in cSCC 

Male sex is a risk factor for cSCC. Male patients are often younger, present more frequently with metastatic disease and have a worse prognosis [138–141]. The increased incidence is often attributed life-style choices. However, more recent research suggests an underlying biological cause [139, 142]. Some scarce evidence infers a role of hormonal signalling in modulating EMT in cSCC. Chen et al. [42] reported estrogen-dependent activation of the FN1-STAT3 axis in the vulva-derived A431 cell line. The subsequently induced EMT could be reversed with the inverse agonist XCT790. Nappi et al. [143] reported on a link between thyroid hormones, EMT, and tumor stage. NANOG and Deiodinase 2 (D2) were proportionately increased significantly with increased cSCC stage. D2 catalyzes the conversion of the thyroid hormone (TH) T4 to T3. TH depletion of the growth medium and inhibition of D2 with rT3 both reverted the EMT phenotype in SCC13 cells. A second study confirmed that T3-induced thyroid hormone receptor α (THR) directly binds the Zeb1 promotor and induces transcription of the EMT-TF [144]. Additionally, high D2 levels correlated with more advanced stage, a higher risk of relapse and lower overall survival in two independent datasets [144]. Together, these findings warrant a closer investigation of the hormonal influence on EMT in cSCC.

#### EMT in cSCC can be attenuated by targeting MAPK, cytokine, growth factor, and NFkB signalling

**Fig. 4:**
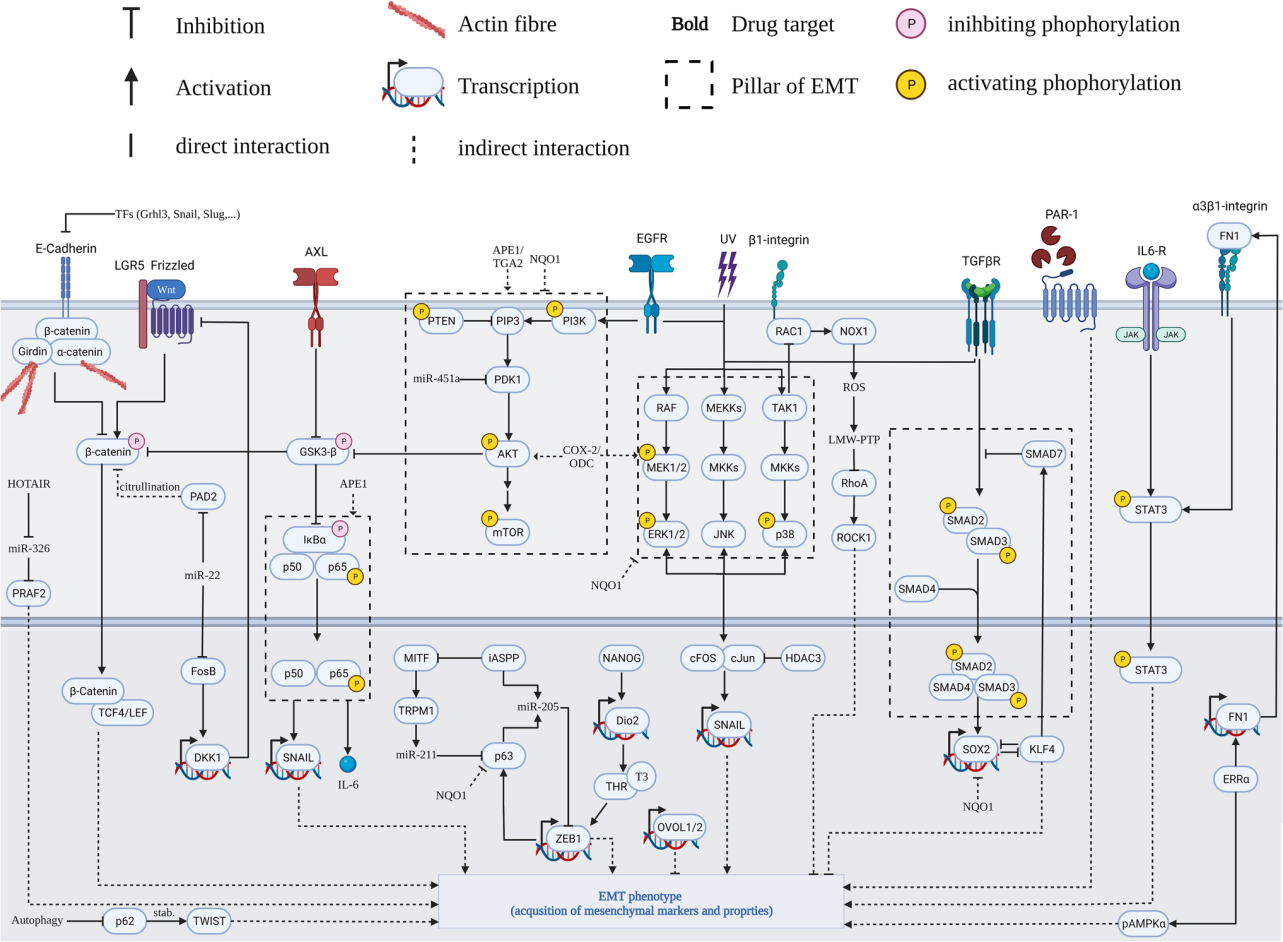
Synthesis of EMT signalling in cSCC based on clinical and experimental studies evaluated in this review (Supplementary Table 1 and 2). The signalling investigated by cell line-based studies was merged in this EMT pathway map under the assumption of transferability between the different aetiologies and models. Multiple changes can induce EMT in cSCC. Hormonal, cytokine, growth factor, ECM signalling all contribute cooperatively to the extent and nature of the EMT programme. Central signalling hubs such as Akt, MKKs or NFκB are attractive drug targets that could be used to attenuate EMT in cSCC [76]

Drug resistance and recurrence are two major challenges modern cancer therapy has to overcome. Due to its link to both these phenomena, the modulation of EMT towards an epithelial phenotype has become a desirable therapeutic avenue [6]. The development of therapy inducing MET would overcome some of these challenges. During past research efforts, some drugs have elicited the desired reversion of an EMT phenotype in cSCC cell lines and xenografts (Table 2). Successful induction of MET was mostly achieved by targeting four major signalling pathways responsible for the induction EMT in cSCC: EGFR signalling, the PI3K/Akt/mTOR pathway, TGF-β signalling and NFκB signalling (refer to Fig. 4 for details). The kinase, Akt, takes a central role here with several drugs, reducing its activity also favourably modulating EMT marker expression, reducing migratory and invasive properties. Direct pharmaceutical inhibition of Akt successfully induced apoptosis, reduced tumor growth in xenografts whilst inducing MET [110, 145]. Inhibition of Cyclooxygenase 2 (COX-2) or Ornithine decarboxylase (ODC) with diclofenac and difluoromethylornithine (DFMO), respectively, reduced p-Akt levels and in single agent and combination therapy [146]. Modulators of other upstream signalling of Akt like EGFR and PI3K-signalling show promise as potential targets [124, 147–149]. Inhibition of the effectors of EGF and TGF-β induced signalling such as p38, JNK, AP1, MEK, and SMAD3 had a similar effect to inhibition of Akt [42, 87, 145, 150]. Tightly connected to p38 signalling is RelA, a component of the NFκB transcription factor. Induction of p-p38 lead to decreases in p-RelA and abrogation of migration, spheroid formation and stemness [108]. Conversely, stabilization of IκBα, a negative modulator of the NFκB pathway, increased cellular adhesion and lead to loss of migration, spheroid formation and stemness [107].

**Table 2:** Drugs and their effects on EMT markers and properties in cSCC

Drug	Target	Markers and signalling	Properties	Model	Refs.
SB431542	TGFBRI	*p-p38, pERK1/2*	*Migration, invasion, ***proliferation**	RDEB-cSCC	[87]
SB203580	p38	*Vim, FN1, MMP9, PAI-1*	*Migration, invasion*	RDEB-cSCC	[87]
Trametinib	MEK1/2	*Vim, FN1, MMP9*	*Migration, invasion*	RDEB-cSCC	[87]
PD169316	SMAD3	*pSMAD3, PAI-1, MMP2, MMP9*	*Migration, invasion*	RDEB-cSCC	[87]
ARP100	MMP2	*MMP2, PAI-1*	*Migration, invasion*	RDEB-cSCC	[87]
Avicularin	n.s	**E-cad**, *N-cad, MMP9, Vim, pMEK, p-p65*	**Apoptosis**	SCC13	[152]
Aloeemodin, Kaempferitrin	EphB2	**E-cad**, *EphB2, MMP9, MMP2, Vim*	*Proliferation, invasion, xenograft growth* **apoptosis**	A431, SCL-1	[154]
Wogonoside	n.s	**E-cad**, *N-Cad, FN1, VEGF, MMP9, MMP14, p-PI3K, p-WNT, p-β-cat, p-AKT*	*Viability, colony formation, stemness, proliferation, invasion, microtubule formation, xenograft growth*, **apoptosis**	SCL-1, SCC12	[112]
rT3	Dio2	**E-Cad**, *N-Cad, Vim*, Zeb1	*Migration*	SCC13	[43]
Ginsenoside®- Rg3	HDAC3	**E-Cad**, *N-Cad, Vim, Snail, HDAC3, c-Jun*	*Migration, invasion*	A431, SCC12	[155]
LY2109761	TGFBRI/II	**E-Cad**, *pSMAD2/3, Vim, FN1, Slug*	*Migration, invasion*	SCL-1	[156]
XTC790	ERRα	**E-Cad**, *p53, FN1, Vim, pSTAT3, pATR, pAMPKα*	*Proliferation, migration, ****apoptosis***	A431	[42]
Niclosamide	STAT3	**E-Cad**, *pSTAT3, FN1, Vim*	n.s	A431	[42]
Lapatinib	HER2/ EGFR	**PTEN, p-PTEN, E-Cad**, *pAKT, p-mTOR, Wnt, β-catenin, N-cad, Vim, Slug*	**Apoptosis, autophagy**, viability	A431	[147]
LY294002	PI3K	**E-Cad**, *pAKT, Vim*	*Migration, invasion*	As-transformed HaCaT	[124]
NC9	TG2	**E-Cad**, *Twist, Snail, Slug, Vim, FN1, N-Cad, HIF1α*	*Spheroid formation, migration, invasion*	A431, SCC13	[110]
Akt inhibitor VIII	Akt	**E-Cad**, *Vim, Slug, pAkt*	***Adhesion****, migration*	PM1, MET1, MET4	[56]
Caffeic Acid	Fyn Kinase	**E-Cad**, *N-Cad, Vim, Snail, p-p38, p-RelA*	*Migration, stemness, spheroid formation*	As-transformed HaCaT	[108]
SB203580	p38	**E-Cad***, N-Cad, Vim, Snail, ***p-p38***, p-RelA*	*Migration, stemness, spheroid formation*	As-transformed HaCaT	[108]
BAY 11–7082	p-IκBα	**E-Cad**, *N-Cad, Vim, Snail*	**Adhesion***, stemness, spheroid formation,* *tumor formation*	As-transformed HaCaT	[107]
Y27632	RhoA	*E-Cad*, **Vim, N-Cad, Snail, Slug**	n.s	A5RT3	[151]
Diclofenac	COX-2	*p-AKT, p-ERK1/2, p-MAPKAP2, Snail, Twist, MMP-2, COX-2*	*Tumor growth, migration, colony formation*, **apoptosis**,	A431 (Xenograft)	[146]
DMFO	OCD	*p-AKT, p-ERK1/2, p-MAPKAP2, ODC*	*Tumor growth, colony formation,* **apoptosis**	A431 (Xenograft)	[146]
Diclofenac and DMFO	COX-2/ OCD	*p-AKT, p-ERK1/2, p-MAPKAP2, Slug, Twist, MMP-9, MMP-2, COX-2, ODC*	Tumor growth, migration, colony formation, **apoptosis**	A431 (Xenograft)	[146]
API-59CJ-Ome	Akt	*p-AKT, Slug, N-Cad, FN1*	*Tumor growth*	A431	[146]
Triciribine	p38	*p-p38, p-MAPKAP2, MMP-2, MMP-9, N-Cad*, **E-Cad**	Tumor growth, proliferation, **apoptosis**	CsA-treated A431 (Xenograft)	[145]
SB-203580	Akt	*p-AKT, pmTOR, p-p38, p-MAPKAP2, MMP-2, MMP-9, N-Cad, ***E-Cad**	*Tumor growth, proliferation*, **apoptosis**	CsA-treated A431 (Xenograft)	[145]
Triciribine/ SB-203580	p38/ Akt	*p-AKT, pmTOR, p-p38, p-MAPKAP2, MMP-2, MMP-9, N-Cad*, **E-Cad**	*Tumor growth, proliferation*, **apoptosis**	CsA-treated A431 (Xenograft)	[145]
luteolin	n.s	*FN1, Vim, Twist, Snail, N-Cad, MMP-9, p-Akt, p-GSK3β, ***E-Cad**	*Migration, invasion*	A431	[153]
Quercetin	n.s	*FN1, Vim, Twist, Snail, N-Cad, MMP-9, p-Akt, p-GSK3β*, **E-Cad**	*Migration, invasion*	A431	[153]
Wortmannin	PI3K	*p-Akt, Vim, ***E-Cad**	n.s	A431	[148]
GSP	n.s	*EGFR, p-ERK1/2, N-Cad, FN1, Vim, ***E-Cad**	*Migration, invasion*	SCC13	[149]
Erlotinib	EGFR	*N-Cad, FN1, Vim*, **E-Cad**	*Invasion*	SCC13	[149]
UO126	MEK	*p-p38, p-ERK, Vim*, **E-Cad**	n.s	Transformed HaCaT (II-3 and H375)	[150]
SP600125	JNK	*Vim*, **E-Cad**	n.s	Transformed HaCaT (II-3 and H375)	[150]
[6]-Gingerol	AP1	*Vim*, **E-Cad**	n.s	Transformed HaCaT (II-3 and H375)	[150]

Italics : attenuated; bold : induced; n.s.: not specified; GSP: grape seed proanthocyanidins

Most compounds tested targeted the aforementioned four major pillars of EMT signalling. However, there are a few exceptions involving hormonal signal transduction and cytoskeletal signalling. XTC790, a reverse agonist for the nuclear estrogen-related receptor α (ERRα), and rT3, a Dio2 inhibitor, elicited responses consistent with MET in A431 and SCC13 cell, respectively [42, 143]. Interestingly, inhibition of RhoA, a small GTPase involved in integrin and cell skeletal signalling, had the opposite effect of all previously discussed drugs and in fact promoted EMT [151]. Additionally, proof of concept studies using several natural compounds have reported modulation of EMT markers and properties in cSCC cell lines [112, 149, 152, 153]. However, these studies lack proper validation of their targets, an in-depth assessment of potential off-target activities, and consequently an understanding for the signalling pathways modulated. For example, Wogonoside modulates p-PI3K, p-β-catenin and p-Wnt levels leaving serious doubts about its selectivity especially given the lack of a specific target in the study [112]. Nevertheless, investigations into the suggested targets, EphB2 and HDAC3, using selective inhibitors might prove fruitful given their implications in other cancers [154, 155].

## Discussion

During the clinical progression from sun-exposed skin to metastatic cSCC, keratinocytes acquire an increasing number of traits consistent with EMT [46, 51, 56] (refer to Fig. 2). The increasing invasiveness paired with changes towards a spindle-shape morphology and altered expression of markers are strong indicators for EMT. The evidence presented confirms that in cSCC the acquisition of advanced EMT features represents the point of transition from benign to malignant, non-invasive to invasive disease. A major difference between differentiated and classical pathway of cSCC progression is the aggressiveness of the arising tumors as well as the point during the progression where tumor cells displaying advanced EMT markers (Vimentin, Zeb1) can be observed [51, 52]. During the differentiated pathway, the point of acquiring invasive capabilities coincides with the expression of advanced EMT markers. During the classical pathway, the EMT transcription factors Snail, Twist and Slug are readily expressed in the pre-neoplastic lesions [46]. In situ cSCC (Bowen’s disease) display signs of early EMT as well as stain positive for the expression of OVOL-transcription factors (OVOL-TFs), negative modulators of EMT [50, 53]. Bowen’s disease acquiring de novo invasive capacity shows more advanced EMT markers at the invasive front such as increased Vimentin [53]. The OVOL-TFs antagonize late-stage TFs such as ZEB1, hence suggesting that in situ cSCC is confined to the epidermal layer due to the inhibition of late stage EMT by the OVOL-TFs [49, 50, 71]. Finally, the clinical extreme of cSCC, sc-SCC, can also be explained by EMT involvement and is plausible given that EMT involvement in the formation of scSCC has been shown for a closely related cancer of cSCC, scSCC- of the head and neck [74, 157–159].

### Akt regulation paralleled by activated TGFβ signalling is central in the regulation of EMT in cSCC

Clinical studies identified the increased activation of Akt, overexpression of EGFR and the presence of active nuclear IκKβ as predictors of aggressive and metastatic disease [56, 160, 161]. Confirming these findings, in vitro studies identify the canonical EGFR/MAPK-pathway, the PI3K/Akt-pathway, and the NFκB-pathway as the central pillars of EMT regulation in cSCC (Fig. 4). Akt can be activated via PI3K and PDK1 by mitogen receptors such as EGFR [162]. Another important hub, GSK3-β, connects Akt signalling with NFκB signalling as well as stemness signalling through stabilization of β-catenin [163, 164].

The fourth pillar of EMT (Fig. 4), canonical TGF-β signalling, was described in an early study by Davies et al. [150]. The minimal requirement of the immortal keratinocyte cell line, HaCaT, to undergo EMT is a combination treatment of EGF and TGF-β. The activation of EGFR signalling can be mimicked by transfection with mutant Ras. Constitutively active Ras was able to activate MAPK signalling which is required to potentiate SMAD-dependent activation of AP1 family TFs. AP1-mediated EMT was an observation that Ji et al. [15] should repeat. Since then TGF-β has been shown to contribute to the induction of EMT via autocrine signalling, paracrine signalling, as well as the induction of an EMT-favouring CAF phenotype. Additionally, suppressors of aberrant canonical SMAD-dependent TGF-β signalling such as SMAD4 and KLF4 are lost during cSCC pathogenesis [64, 66]. TGF-β and underlying SMAD, ERK and P38 signalling induces the transcription of multiple EMT related proteins such as COX-2, Snail, Slug, proteases (MMP-2, MMP-9) and SERPINE1 [47, 156, 165]. An overview over the complex signalling circuity underlying EMT and the crosstalk between the four pillars is provided in Fig. 4.

### EMT-targeting therapeutics might be valuable in adjuvant therapies for high-risk tumors and immunotherapy

Immunosuppression is a major risk factor for cSCC [23]. In murine models of cSCC, cyclosporine A-mediated repression of immunosurveillance gave rise to aggressive tumors- bearing a strong EMT signature [166]. Similar observations in humans also point towards a potential application of EMT-targeting therapeutics to mitigate malignant transformation in immunosuppressed patients [167]. Additionally, EMT markers have been linked to resistance against immunotherapy, an emerging treatment for inoperable advanced and metastatic cSCC [7, 15, 167]. EMT itself and the closely linked stem-like properties have been linked to drug resistance and recurrence in multiple cancers [6]. Consequently, reducing EMT and associated stemness might prove useful in assisting current and future therapeutics to overcome these challenges. As shown in Table 2, targeting canonical EGFR, canonical TGF-βR, NFκB, and PI3K-signalling successfully reverse EMT. Within in these pathways, connecting hubs such as AKT pose interesting drug targets (Fig. 4). Downstream effector kinases such as ERK or p38 can also be used to attenuate EMT and prevent the transcriptional induction of EMT-TFs.

However, the targeted treatments used in the past are merely valuable proof-of-concept studies due to their insufficient drug properties for clinical translation. Lacking selectivity, unclear mechanism of action, or a poor side effect profile are only a few of the problems that need to be addressed. However, these studies can serve as part of the target validation process or as lead for the development of more selective compounds. Replacing compounds with current alternatives that have possibly undergone pre-clinical and clinical testing or are approved by the respective governing body can be a valid strategy to assess their efficacy against cSCC.

In the future, therapeutic strategies could be amended by new targets and drug classes such as miRNA therapeutics that replace tumor suppressor miRNAs lost during progression or targeting EMT-TF directly [168]. With increasing advancements in RNA-based technologies, ectopic OVOL-TF induction might become feasible [169]. Modulation of p62 levels to attenuate EMT and subsequent induction of apoptosis via an autophagy-dependent mechanism is an interesting mechanism [136]. Inhibitors of NFκB or Wnt/βcatenin signalling could attenuate the EMT-linked stemness and EMT and hence help mitigate -associated complications such as therapy resistance. Hence, increasing research in small molecule and peptide inhibitors makes targeting key protein–protein such as SMAD interactions or the dimeric NFκB transcription factor become more attainable [170].

The uPA system is a regulator of MMPs and hence might be an attractive therapeutic target to reduce invasion and metastasis. The upregulation of uPA/ uPAR and SERPINE1 is associated with poor prognosis in multiple cancers including HNSCC and many other solid tumors [171–174]. uPA-uPAR complexes can interact with integrins, vitronectin and LDLR endocytosis receptors as well as induce plasmin-mediated ECM degradation [171–174]. PAI-1 modulates cell adhesion and migration via competing with integrins and uPAR for vitronectin binding sites and can act as co-receptor for LDLRs and convey mitogenic signalling [172, 174–176] High levels of both uPAS components and PAI-1 might also be required for the precise spatial and temporal regulation of tumor cell invasion and focalized ECM remodelling [177, 178]. Additionally, amiloride derivatives, a class of uPA inhibitors, have shown some success in murine models completely preventing metastasis in an aggressive pancreatic cancer model [179, 180]. The induction of uPA activity by specific combination of EGF and TGF-β1 treatment in HaCaT derivatives alongside of induction of EMT implies a strong link between the regulation of uPAS and the regulation of EMT in keratinocytes [15, 118]. Some clinical evidence supports the exploration of uPA as a therapeutic target. Minaei et al. [181] report the significant upregulation of *PLAU* (uPA) and *PLAUR* (uPA receptor) in metastatic tumors vs. non-metastatic primary cSCCs.

### EMT markers can aid in tumor risk stratification and are linked functionally to invasion and metastasis

In multiple cancers, EMT markers are found on cells with invasive capacities and on circulating tumor cells [10, 14, 15]. This links EMT conceptually to metastasis. In cSCC, EMT markers also correlate with increased metastatic risk, increased progression, decreased recurrence and increased disease-specific death [54, 56, 151]. Barrette et al. [56] and Toll et al. [58] linked EMT markers to invasion and metastasis in cSCC. Additional biomarkers for aggressive and metastatic disease include the indicators of increased signalling of two additional pillars, the overexpression of EGFR and the presence of active nuclear IκKβ [160, 161]. This warrants further investigation into an EMT-marker based risk stratification of primary tumors aiming to predict metastasis and guide clinical interventions. This would put cSCC in the line of cancers that push for clinical adaptation of EMT markers for risk stratification as well as meeting a desperate need for metastasis markers in cSCC [18, 182–184].

## Conclusion

EMT plays a pivotal role in the clinical progression of cSCC. EMT-markers are associated with worse patient outcome and recurrence. This makes EMT a desirable process to target using small molecule inhibitors and other therapeutics. Potential therapeutic targets include members of the driving signalling pathways (EGFR, NFκB, TGF-β, PI3K) and effectors that execute changes such as ECM degradation (uPA). Modulating EMT or mitigating its effects has the potential to become a powerful therapeutic approach to assist current therapies such as immunotherapy as well as targeted therapies in development.

## Supplementary Information

Below is the link to the electronic supplementary material.

Supplementary file1 (PDF 498 KB)

Supplementary file2 (PDF 399 KB)

Additional file3 (XLSX 27 KB)

Additional file4 (XLSX 33 KB)

## Abbreviations

EMT: Epithelial-mesenchymal transition
TME: Tumor microenvironment
UV: Ultraviolet
cSCC: Cutaneous squamous cell carcinoma
RDEB: Recessive dystrophic epidermolysis bullosa
AK: Actinic keratosis
lacSCC/mcSCC: Locally advanced/metastatic cSCC
EMT-TF: Epithelial-mesenchymal transition transcription factor
TEMTIA: The epithelial-mesenchymal transition international association
SES: Sun exposed skin
PNI: Perineural invasion
TSK: Tumor specific keratinocyte
sc-cSCC: Spindle-cell cSCC
SCC: Cutaneous Carcinosarcoma
CAF: Cancer-associated fibroblast
MMP: Matrix metalloprotease
uPA/uPAR/uPAS: Urokinase plasminogen activator/uPA receptor/uPA system
ECM: Extracellular matrix
CSC: Cancer stem cell
