# Supplementary material for: A tEMTing target? Clinical and experimental evidence for epithelial-mesenchymal transition in the progression of cutaneous squamous cell carcinoma (a scoping systematic review)
Source: Discov Oncol. 2022 Jun 6;13:42. doi: 10.1007/s12672-022-00510-4 (PMC9170863; doi:10.1007/s12672-022-00510-4)
Supplement: Supplementary file 2 — Supplementary file2 (PDF 399 KB) [file 12672_2022_510_MOESM2_ESM.pdf]

Supplementary File 2: Search Strategy

Date	Database	Search String	# Results	Filters
26/10/21	Scopus	TITLE-ABS-KEY((KEY OR {Epithelial Mesenchymal Transition} OR {Cell plasticity} OR {single cell}) AND ({skin cancer} OR {skin neoplasm} OR {skin carcinoma} OR {keratinocyte carcinoma} OR {cutaneous squamous cell carcinoma} OR {cSCC}))	2192	none
26/10/21	Web of Science	TI=((“Epithelial Mesenchymal Transition” OR “Cell plasticity” OR “single cell”) AND (“skin cancer” OR “skin neoplasm” OR “skin carcinoma” OR “keratinocyte carcinoma” OR “cutaneous squamous cell carcinoma” OR “cSCC”)) OR AB=((“Epithelial Mesenchymal Transition” OR “Cell plasticity” OR “single cell”) AND (“skin cancer” OR “skin neoplasm” OR “skin carcinoma” OR “keratinocyte carcinoma” OR “cutaneous squamous cell carcinoma” OR “cSCC”)) OR AK=((“Epithelial Mesenchymal Transition” OR “Cell plasticity” OR “single cell”) AND (“skin cancer” OR “skin neoplasm” OR “skin carcinoma” OR “keratinocyte carcinoma” OR “cutaneous squamous cell carcinoma” OR “cSCC”))	141	none
26/10/21	PubMed	((Epithelial Mesenchymal Transition [MeSH Terms]) OR (Cell plasticity [MeSH Terms]) OR “Epithelial Mesenchymal Transition” OR “Cell plasticity” OR “single cell”) AND (“skin cancer” OR “skin neoplasm” OR “skin carcinoma” OR “keratinocyte carcinoma” OR “cutaneous squamous cell carcinoma” OR “cSCC” OR (Skin neoplasms [MeSH Terms]))	575	none
26/10/21	Medline	((MH "Epithelial Mesenchymal Transition") OR (MH "Cell plasticity") OR “Epithelial Mesenchymal Transition” OR “Cell plasticity” OR “single cell”) AND (“skin cancer” OR “skin neoplasm” OR “skin carcinoma” OR “keratinocyte carcinoma” OR “cutaneous squamous cell carcinoma” OR “cSCC” OR (MH "Skin neoplasms"))	555	none

A tEMTing target? - Clinical and Experimental Evidence for Epithelial-Mesenchymal Transition in the Progression of Cutaneous Squamous Cell Carcinoma (A Scoping Systematic Review) in Cancer and Metastasis Reviews by Benjamin Genenger [1,2], Jay R. Perry [1,2], Bruce Ashford [2,3], Marie Ranson [1,2]

1. School of Chemistry and Molecular Bioscience, University of Wollongong, Wollongong, NSW, Australia

2. Illawarra Health and Medical Research Institute, Wollongong, NSW, Australia

3. School of Medicine, University of Wollongong, Wollongong, NSW, Australia

Corresponding Authors: Benjamin Genenger (bg038@uowmail.edu.au) and Marie Ranson (mranson@uow.edu.au)
